# Supplementary material for: Effect of Growth Factors and Hormones during In Vitro Growth Culture of Cumulus-Oocyte-Complexes Derived from Small Antral Follicles in Pigs
Source: Animals (Basel). 2023 Mar 30;13(7):1206. doi: 10.3390/ani13071206 (PMC10093161; doi:10.3390/ani13071206)
Supplement: Supplementary file 1 [file animals-13-01206-s001.zip › animals-2251273-supplementary.pdf]

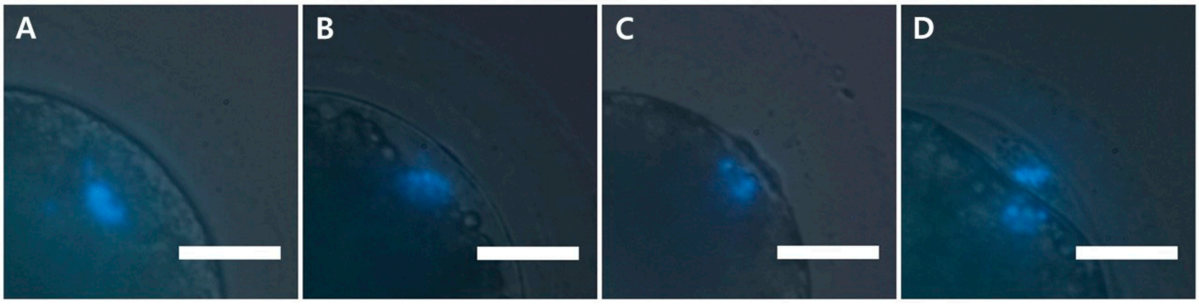

Figure S1. Nuclear status of porcine oocytes. Stained with Hoechst 33342: (A) GV/GVBD; (B) MI; (C) AI/TI; and (D) MII. Scale bar = 20 μm.

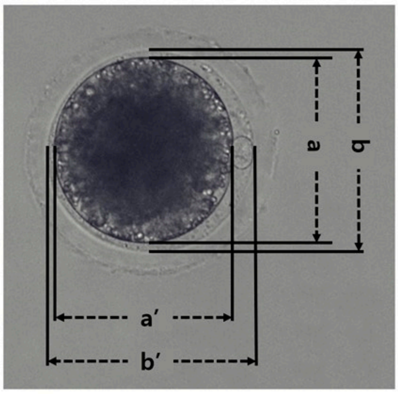

Dimeter of oocyte (A) = (a+a′)/2

Inner dimeter of zona pellucida (B) = (b+b′)/2

Size of perivitelline space = (B-A)/2

Figure S2. Diagram of the measurement of oocyte diameter, and the size of the perivitelline space in porcine oocytes.
